# Lateralisation of language and emotion in schizotypal personality: Evidence from dichotic listening

**DOI:** 10.1016/j.paid.2011.06.017

**Published:** 2011-10

**Authors:** Antonio Castro, Rebecca Pearson

**Affiliations:** Psychology Division, School of Social Sciences, Nottingham Trent University, Burton Street, Nottingham NG1 4BU, United Kingdom

**Keywords:** Hemispheric laterality, Emotion recognition, Word detection, Schizotypal personality, Dichotic listening

## Abstract

Striking disturbances have been reported in language and emotional prosody processing by patients diagnosed with schizophrenia. In view of this and of research suggesting that schizotypal personality traits can also be expressed sub-clinically, the present study aimed to discover whether similar disturbances would be reflected in cognitive laterality patterns when symptoms of schizotypy are present yet at a non-clinical level. A dichotic listening task was used to examine the sensitivity and speed with which 132 right-handed participants (85 females and 47 males, mean age = 32.44, *SD *= 12.29) detected both words and emotional prosody, all of whom also completed the Schizotypal Personality Questionnaire. Findings indicated that both high (*n *= 64) and low (*n *= 68) schizotypy groups demonstrated the typical right ear advantage for the detection of words and left ear advantage for the detection of emotional prosody. Individuals with higher schizotypal personality scores also demonstrated poorer sensitivity in detecting emotional prosody. These results reveal that within the healthy population, higher levels of schizotypy are not associated with the atypical lateralisation of language and emotion. Nevertheless, the existence of these symptoms does signal the presence of shared characteristics with the clinical sphere, namely poorer emotion recognition performance.

## Highlights

► High and low schizotypy groups showed right ear advantage for detecting words. ► Both schizotypy groups showed left ear advantage for detecting emotional prosody. ► High schizotypy subjects displayed poorer sensitivity in detecting emotional prosody.

## 1 Introduction

In healthy right-handed individuals, the left hemisphere tends to be better at processing fundamental aspects of language such as phonemes and syntax, whereas the right hemisphere specialises in the perception of emotional prosody (Bryden & MacRae, 1988). However, patients suffering from psychiatric illnesses frequently demonstrate impaired performance on dichotic listening measures of hemispheric asymmetry (Sommer, Ramsey, & Kahn, 2001). Specifically, a reduction in, or complete absence of the expected right ear advantage (REA) for linguistic stimuli has been observed in schizophrenia (Green, Hugdahl, & Mitchell, 1994). This decrease in REA has been found not to be associated with cognitive performance (Sakuma, Hoff, & DeLisi, 1996), but with positive clinical symptoms such as hallucinations (Bruder et al., 1995). Patients diagnosed with schizophrenia have also shown deficits in emotion recognition linked to reduced right hemisphere lateralisation (Ross et al., 2001). These results have led previous researchers (e.g., Edgar et al., 2006) to maintain that atypical hemispheric asymmetries could reflect a general risk factor associated with psychiatric illness.

Accumulating research has also documented the prevalence of schizotypal traits among non-clinical populations (Johns & van Os, 2001; Siever & Davis, 2004). In schizotypy, for instance, which is a set of personality characteristics and experiences that indicate the degree of predisposition to schizophrenia, the role of the left hemisphere in language processing has been explored using a variety of cognitive tasks (e.g., Overby, 1992; Suzuki & Usher, 2009). These studies have frequently revealed a left hemisphere dysfunction in high schizotypal participants similar to, but less severe than those recognised in schizophrenia. Specifically, reduced lateralisation of language, suggestive of an underactive left hemisphere, has been reported (Rawlings & Borge, 1987; Suzuki & Usher, 2009). Similarities with schizophrenia have also been observed in the way of a complete reversal of the expected lateralisation in favour of increased right hemisphere performance (Rawlings & Claridge, 1984). Schizotypal individuals have even demonstrated overactivation of the left hemisphere when processing linguistic information (Overby, 1992). Whilst this slight over-activation produces superior performance, greater activation can lead to a dysfunctional state and impaired performance.

The disparity present in these findings may be attributed to the variety of stimuli utilized across the measures of lateralisation. Employing the divided visual field technique, which involves presentation of visual stimuli to either the left or right visual field, Broks (1984) and Suzuki and Usher (2009) demonstrated reduced left hemisphere specialisation of language with consonant–vowel–consonant nonsense syllables. Operating within the same sensory modality, Rawlings and Claridge (1984) demonstrated a reversal of the expected left hemispheric dominance for language, in favour of superior right hemisphere performance. This result of a right hemisphere specialisation may be due to the utilisation of letters as stimuli, which can be recognised using two strategies. Specifically, the authors suggest that two personality types might rely on different processing mechanisms, with the high schizotypy group possessing a visual processing skill (implicating the right hemisphere), compared to the low schizotypy group who utilize the typical linguistic strategy (implicating the left hemisphere). Despite these heterogeneous findings, it appears that commonalities exist between schizophrenia and the sub-clinical level of the schizotypal personality spectrum in the way of lateralisation for language. These commonalities may be influenced by the number and severity of some of the symptoms experienced. Sommer and collaborators (2001), for example, found that patients suffering from schizophrenia who had less severe hallucinatory symptoms, displayed an increased language lateralisation that pointed towards the typical laterality pattern of control subjects. Therefore, it remains to be elucidated whether the laterality patterns of non-clinical schizotypy individuals are in line with those observed in a healthy population, or those observed in patients with schizophrenia.

In an attempt to examine the contribution of both hemispheres to language processing within this population, Nunn and Peters (2001) employed a range of tasks that assess the linguistic abilities of both the left and right hemispheres. Findings revealed that right hemisphere dysfunction was the main predictor of high schizotypy within the non-clinical sample. Thus, it appears that in line with schizophrenia, dysfunctions of both hemispheres are present in schizotypy. Despite this right hemisphere deficit, lateralisation of emotion has seldom been studied within this population. The paucity of research in this domain becomes even more surprising in view of numerous reports of emotion recognition impairments in schizotypy (Aguirre, Sergi, & Levy, 2008; Phillips & Seidman, 2008). These deficits in both facial and prosodic emotion recognition are analogous to those impairments observed in schizophrenia, which are believed to be a consequence of reduced right hemisphere lateralisation (Ross et al., 2001). It is possible that healthy individuals experiencing schizotypy traits may also demonstrate dysfunctional emotional processing, comparable to those observed in schizophrenia (Edwards, Jackson, & Pattison, 2002). This is yet to be confirmed as, of those studies employing emotional recognition tasks (e.g., Aguirre et al., 2008; Toomey & Schuldberg, 1995), the hemispheres’ contribution to the processing of emotional prosody has not been examined in schizotypy.

In light of this research, it is evident that the current understanding of hemispheric responses to language and emotional prosody at the sub-clinical level of the schizotypal personality spectrum are inconclusive. Specifically, it remains unclear whether healthy individuals who may experience signs and symptoms present in schizotypal personality but do not qualify for clinical diagnosis, display the laterality patterns characteristic of healthy individuals, or resemble the atypical laterality observed within schizophrenia. The current understanding of the left hemisphere’s role in language processing is ambiguous and findings indicate that symptomatology as well as symptom severity may influence laterality patterns (Bleich-Cohen, Hendler, Kotler, & Strous, 2009; Sommer et al., 2001). Moreover, the right hemisphere’s role in emotional prosody processing within a non-clinical sample is still unknown. Nevertheless, findings of emotion recognition deficits in this population (e.g., Phillips & Seidman, 2008), suggest that impaired emotion perception, akin to language deficits, appears to be related to unusual lateralisation. Considering the prominent contributions of each of the hemispheres to speech comprehension and in view of current findings in this area in the schizotypal personality spectrum, the need for further investigation at a sub-clinical level is warranted.

In order to re-examine language lateralisation at the sub-clinical level, while simultaneously investigating the lateralisation of emotional prosody processing, the current study employed the dichotic listening paradigm developed by Bryden and MacRae (1988). It was hypothesised that individuals who score low in schizotypal personality traits would demonstrate the expected REA for the perception of words and left ear advantage (LEA) for the perception of emotional voice tones. In view of the nature of schizotypal personality, combined with previous reports of atypical linguistic processing and emotional recognition deficits; the present study aimed to determine whether the laterality patterns of high schizotypy participants reflect those characteristic of a healthy population, or those frequently reported within the clinical sphere.

## 2 Method

### 2.1 Participants

A total of 132 healthy adults (47 males and 85 females; mean age = 32.44 years, *SD *= 12.29) were recruited from Nottingham Trent University. All participants reported to be native English speaking, right-handed, which was confirmed by the Edinburgh Inventory (Oldfield, 1971), and had no hearing deficits. Additional item measures were taken to screen for and exclude any individuals that were currently suffering from, or reported any previous history of neurological conditions, psychiatric illnesses or impaired language ability. The sample was divided into high (*n *= 64) and low (*n *= 68) schizotypal personality groups by the median of the total Schizotypal Personality Questionnaire (SPQ) score (median = 17; range, 1–46; see Table 1). This approach allowed for the assessment of range-bound schizotypy effects and has previously been used elsewhere (e.g., Hori, Ozeki, Terada, & Kunugi, 2008; Langdon & Coltheart, 2004). No significant differences in demographic variables were found between the two groups, indicating equal dispersions of sex [*X*^2^ (1, *N *= 132) = 067, *p* > .05] and age [*t*(119) = 1.48, *p* > .05]. In addition, all participants were treated in accordance with the Declaration of Helsinki (International Committee of Medical Journal Editors, 1991).

### 2.2 Materials

#### 2.2.1 Experimental task

The auditory stimuli used within the present dichotic listening task consisted of four words (‘dower’, ‘tower’, ‘power’, and ‘bower’), each pronounced in four different emotional tones (happy, sad, angry, and neutral), resulting in 16 separate word–emotion combinations. These were spoken by an adult male and recorded using a digital recorder. After the stimuli were obtained, they were edited to a common length of 560 ms and equalised in loudness.

Originally four versions of each word–emotion combination were gathered, totalling 64 recordings. After editing, these stimuli were presented to a group of 4 participants who were asked to report the word and emotional tone and to rate the intensity (on a scale of 1–5) with which it was spoken. From this, the final stimuli were constructed by selecting the 16 word–emotion sound files that were most correctly identified. To ensure that these 16 recordings were perceived accurately, an additional ten participants were asked to report each word and emotional tone. The emotions were recognised with a minimum accuracy of 69% (*M *= 81.4) and words were identified with a minimum accuracy of 94% (*M = *98.8). Following confirmation of the stimuli, all potential pairings of word–emotion combinations were created, generating 144 stimulus pairs in total. These stimuli were presented over headphones and the experiment was run on SuperLab software.

#### 2.2.2 The Edinburgh Handedness Inventory (EHI; Oldfield, 1971)

This 10-item scale requires participants to specify their hand preference for 10 activities including writing, drawing, throwing, and striking a match. Participants are requested to indicate whether they predominantly use their right hand, left hand, or have no preference. These answers are scored +10, −10, and 0, respectively. Potential scores therefore range from −100 (indicating maximum left-handedness) to +100 (indicating maximum right-handedness). In the present study, all right-handed participants scored at least 60 or above.

#### 2.2.3 Schizotypal Personality Questionnaire (SPQ; Raine, 1991)

This 74-item self-report scale with a “yes/no” response format measures schizotypy traits and features the DSM-III-R (American Psychiatric Association, 1987) criteria for a diagnosis of schizotypal personality disorder (SPD). All items answered “yes” are scored 1 point. According to Raine (1991), the SPQ has demonstrated high internal reliability (Cronbach’s alpha = 0.91), test–retest reliability (*r* = 0.82), and criterion validity (*r* = 0.68 between the SPQ and SPD scores derived from diagnostic interviews).

### 2.3 Procedure

Before hearing the dichotic pairs, participants listened to and familiarised themselves with both the verbal and emotional characteristics of the 16 word–emotion stimuli. A practice session then allowed them to gain experience of the task while receiving feedback on whether responses made were correct or incorrect. The dichotic listening experiment followed (Bryden & MacRae, 1988). Participants were presented with a target word or target emotion on screen at the start of a block of 144 trials and were instructed to monitor for that target. The word targets were ‘tower’ and ‘dower’ and the emotion targets were ‘happy’ and ‘angry’. Participants monitored each of these targets for one complete block, thus there were four blocks of 144 trials totalling 576 trials. During each block the target was present on 50% of the trials; 25% in the right ear and 25% in the left ear.

During a trial, participants heard two sounds simultaneously; one in the right ear and one in the left. Following this stimuli presentation, they indicated if they heard the target in either ear by pressing the green (present) or red (absent) keys of the computer’s response pad. The hand that was used to respond and the target presentation order were both counterbalanced. To allow a space between stimulus presentations, a pause of 700 ms was introduced after individuals responded and before the next sound appeared. A reminder of the target was also presented on the computer screen after every 18 trials. Participants were informed that the aim was to respond as quickly and accurately as possible. Following completion of the experiment, the SPQ and EHI were administered.

### 2.4 Statistical analysis

The current study had a mixed design with two within-subject variables: Task (focus on word, focus on emotion) and Ear (left ear, right ear) in addition to one between-subjects variable: Schizotypal Personality Group, SPQ (high schizotypal personality, low schizotypal personality). Before conducting the statistical analyses, the average number of hits (i.e., correct detections), false alarms (i.e., identifying a target as present when it was absent), and reaction times for hits were computed for each condition. Hit and false alarm rates were employed to calculate *d′*; a signal detection measure of sensitivity that controls for participants’ response bias. Following this, and after univariate assumption testing was complete, a main analysis of variance (ANOVA) was conducted for each one of the two dependent variables; sensitivity (*d’*) and reaction time. Any significant effects were then followed up with post hoc *t*-tests where appropriate.

## 3 Results

### 3.1 *d*′ Analysis

**Fig. 1:**
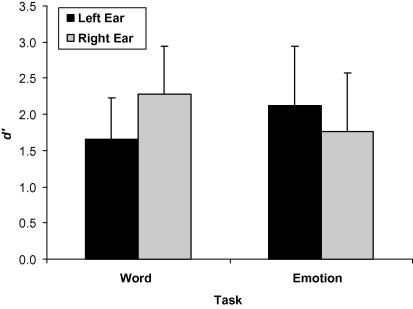
Mean *d*′ values for the detection of word and emotion targets presented to the left and right ears. Error bars represent standard deviation.

Analysis of sensitivity data demonstrated a significant Task × Ear interaction [*F*(1, 130) = 249.16, *p* < .001, $\eta_{p}^{2}$ = .657]. A partial eta squared ($\eta_{p}^{2}$) of .657 indicated that the strength of this relation was large based on Cohen’s (1988) recommendation that small, medium, and large effects are reported as .01, .06, and .14, respectively. The interaction itself showed that participants performed better when words were delivered to the right ear rather than to the left as depicted in Fig. 1 and confirmed by post hoc tests [*t*(132) = −10.21, *p* < .001, $\eta_{p}^{2}$ = .443]. *t*-tests also revealed that participants were more accurate in detecting emotions that were delivered to their left, rather than to their right ear [*t*(132) = 8.07, *p* < .001, $\eta_{p}^{2}$ = .332]. Task × Ear × SPQ did not approach significance, indicating that this typical pattern of laterality was observed across both schizotypy groups [*F*(1, 130) = .08, *p* > .05, $\eta_{p}^{2}$ = .001, see Table 2].

**Fig. 2:**
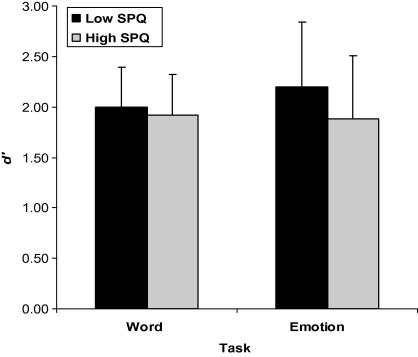
Mean *d*′ values for the detection of word and emotion targets by high and low schizotypal personality groups. Error bars represent standard deviation.

A significant main effect of SPQ [*F*(1, 130) = 8.05, *p* = .005, $\eta_{p}^{2}$ = .058] indicated that discrimination differences exist between the two groups. The low schizotypy group demonstrated higher sensitivity in detecting targets overall [*M *= 2.15, *SD* = .631] compared to the high schizotypy group [*M *= 1.93, *SD* = .615]. Thus, although the high schizotypal personality group displayed typical laterality patterns, its discrimination ability was reduced in relation to the low group. A significant Task × SPQ interaction [*F*(1, 130) = 4.19, *p* = .043, $\eta_{p}^{2}$ = .031] revealed that the low schizotypy group had better discrimination on the ‘emotion’ task than the high schizotypy group [*t*(130) = 2.85, *p* = .005, $\eta_{p}^{2}$ = .059] (see Fig. 2). The partial eta squared reinforces that the magnitude of the difference in mean scores between the groups was small to moderate. In contrast, no significant differences were found between the groups in the ability to accurately detect word targets [*t*(130) = 1.22, *p* > .05, $\eta_{p}^{2}$ = .011]. The low schizotypal personality group also demonstrated more accurate discrimination for ‘emotion’ targets than ‘word’ targets [*t*(67) = −2.66, *p* = .010, $\eta_{p}^{2}$ = .095], whereas the high schizotypy group showed no differences on the performance of these tasks [*t*(63) = .418, *p* > .05, $\eta_{p}^{2}$ = .002].

### 3.2 Reaction time analysis

**Fig. 3:**
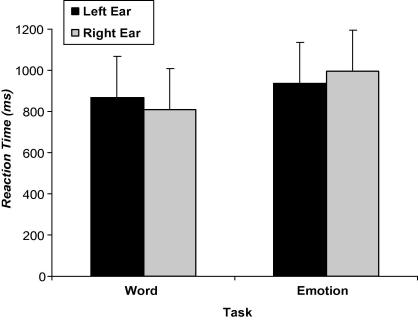
Mean reaction times of correctly detected word and emotion targets presented to the left and right ears. Error bars represent standard deviation.

The analysis of mean reaction time mirrored the significant Task × Ear interaction and the large magnitude of effects [*F*(1, 130) = 62.38, *p* < .001, $\eta_{p}^{2}$ = .324] that were observed in the accuracy data (see Fig. 3). Specifically, reaction times were faster for word targets presented to the right ear [*t*(131) = 5.47, *p* < .001, $\eta_{p}^{2}$ = .186], and for emotion targets presented to the left ear [*t*(131) = −4.58, *p* < .001, $\eta_{p}^{2}$ = .138]. A significant main effect of Task was also found [*F*(1, 130) = 101.90, *p* < .001, $\eta_{p}^{2}$ = .439], signifying faster responses on the word recognition task (*M = *838.30, *SD *= 153.67) than on the emotion task (*M *= 965.67, *SD *= 196.30).

There were no main effects or interactions involving SPQ on reaction time data. In line with the accuracy findings, this indicated that the typical laterality pattern was evident across both high and low schizotypy groups. However, in contrast to the sensitivity data, no significant differences emerged in reaction time between the two groups when they were compared across tasks. Therefore, whereas the low schizotypy group was significantly more accurate at detecting emotions than the high schizotypy group, both groups performed similarly on the amount of time required to detect these targets.

## 4 Discussion

In light of mounting evidence suggesting commonalities between schizophrenia and schizotypy (Siever & Davis, 2004), the primary aim of the current study was to investigate the lateralisation of cerebral responses to words and emotional prosody at the sub-clinical level of the schizotypal personality spectrum. As predicted, healthy individuals with low schizotypal personality scores demonstrated the typical pattern of hemispheric lateralisation on measures of sensitivity and reaction time. This pattern, specifically a REA for the detection of words and a LEA for the detection of emotional prosody, was also observed in individuals who reported higher levels of schizotypy traits. Therefore, atypical hemispheric asymmetry; evident in both schizophrenia and SPD, does not seem to be present at the sub-clinical level of the schizotypy spectrum when using the method and analytic approach used in this study. Despite findings of healthy lateralisation patterns across the sample, sensitivity data did reveal differences in performance between the two groups. In comparison to low scorers, the high schizotypy group exhibited impaired detection of emotional prosody. This suggests that whilst atypical laterality is not a dominant feature of this population, disturbances in emotion recognition do manifest at the high end of the sub-clinical level of the schizotypal personality spectrum.

The demonstration of a left hemisphere specialisation for word detection across measures of sensitivity and reaction time is consistent with, and replicates previous research that has also documented the linguistic proficiency of this hemisphere (Josse & Tzourio-Mazoyer, 2004). Overall, the results did not indicate atypical lateralisation of language; a pattern of hemispheric functioning frequently observed in patients with schizophrenia (Bleich-Cohen, Hendler, Kotler, & Strous, 2009). This is probably due to the severity of symptoms in the low and high SPQ groups. Sommer and collaborators (2001), for example, demonstrated that symptom severity is crucial in determining the extent of anomalous laterality in schizophrenia, with patients who experience more severe symptoms, demonstrating a more atypical pattern of lateralisation. As the present study examined schizotypy at the non-clinical level, it is likely that symptoms at this stage are not severe enough to produce dysfunctional left hemisphere activity. However, previous studies that have also explored language processing at the non-clinical level of schizotypy have yielded mixed results. Many of which, contrary to the present study, have demonstrated atypical language lateralisation in high schizotypal participants, similar to, but less severe than those observed in schizophrenia (Broks, Claridge, Matheson, & Hargreaves, 1984; Overby, 1992; Rawlings & Borge, 1987). Thus, differences in the level of symptoms may not be sufficient in explaining the differences in lateralisation patterns.

A more sophisticated explanation for the discrepancies in findings may be attributable to the specific types of symptoms experienced across the samples. Green and colleagues (1994) argued that in schizophrenia, hallucinations, as opposed to psychotic symptoms in general, are the specific trait that produce impaired performance on dichotic listening measures. The authors propose that this is a result of the left hemisphere attending to inner speech and voices during auditory hallucinations. Further evidence of the significant role that positive symptoms such as hallucinations play in producing atypical laterality was demonstrated by Conn and Posey (2000), who used the dichotic listening paradigm to compare the performance of healthy college students who report verbal hallucinations with college students who report no previous history of this. The authors confirmed that only participants who had reported experiencing auditory hallucinations demonstrated impaired performance, specifically for the detection of words, and thus indicative of left hemisphere dysfunction. The present study tested healthy individuals at the non-clinical level of the schizotypy spectrum who were unlikely to experience hallucinatory symptoms and thus did not demonstrate abnormal lateralisation.

In contrast to the collection of research examining language laterality, this was the first known study to explore hemispheric responses to emotional prosody in non-clinical schizotypy. In line with previous emotion recognition research within this population (Aguirre et al., 2008; Phillips & Seidman, 2008), reduced sensitivity for the detection of emotional prosody was observed within the high schizotypal personality group. As most examinations of emotion perception abilities in schizotypy and schizophrenia tend to focus predominantly on facial affect (Toomey & Schuldberg, 1995), this highlights the importance of investigating prosody, as it appears that impaired emotion recognition is not limited solely to facial affect. Most importantly, however, was the finding of typical right hemisphere specialisation for the detection of emotional tones across the sensitivity and reaction time data. This indicates that poorer sensitivity for detecting emotional prosody in the high schizotypal personality group cannot be attributed to atypical performance of the right hemisphere. The right hemisphere superiority was observed for both positively and negatively valenced words. This better overall performance of the right hemisphere favours the so called ‘right hemisphere hypothesis’ (Borod et al., 1998) over the rival ‘valence hypothesis’, which proposes that the right hemisphere is specialised solely for negative emotions and that positive emotions are processed in the left cerebral hemisphere (Reuter-Lorenz & Davidson, 1981). Premkumar and collaborators (2011) go still further in the study of emotional processing by identifying activational differences between low and high schizotypy in the bilateral dorsal anterior cingulate cortex, right superior frontal gyrus, and left ventral prefrontal cortex when focusing on social rejection as a particular emotion.

However, the present study had a couple of potential limitations that should be noted in generalising from its findings. First, the dichotic listening paradigm used to test hemispheric lateralisation is not nearly as reliable as the Wada test (Woermann et al., 2003), which is taken to be the “gold standard” technique for language lateralisation. However, the Wada test (intracarotid amobarbital hemispheric sedation) has the disadvantage of its invasiveness and the possibility of clinical complications. Additionally, the SPQ range of the sample used in this study, although similar to previous studies (e.g., Langdon & Coltheart, 2004), was relatively low compared to the maximum SPQ range indicated by Raine (1991). This highlights the importance of conducting further research in a more representative community sample.

Taken as a whole, the current findings provide support for the notion that schizotypal personality symptoms are distributed, to varying degrees, throughout the general population of healthy individuals. It can be confirmed that, at a non-clinical level, the presence of these symptoms do not give rise to the atypical lateralisation of language and emotion that is frequently observed within SPD and schizophrenia. Whilst atypical laterality is not a dominant feature of this population, disturbances in emotion recognition do manifest at the high end of the sub-clinical level of the schizotypal personality spectrum. This denotes that overlapping characteristics with the clinical sphere do exist. As the present study provided the first examination into the lateralisation of emotional prosody within this population, it may shed additional light on previous research by confirming that findings of impairments in emotion recognition abilities are unlikely to be a consequence of a right hemisphere abnormality.

## Figures and Tables

**Table 1:** Mean (and SD) of SPQ total scores and subscale scores for both schizotypal personality groups.

SPG	IR	ESA	OB	UPE	EB	NCF	OS	CA	S	TSPQS
Low	0.87 (0.93)	1.81 (1.66)	0.51 (0.91)	0.88 (1.18)	0.81 (1.16)	0.71 (1.05)	2.21 (1.80)	0.76 (0.90)	0.84 (0.96)	9.40 (4.39)

High	3.47 (2.34)	4.19 (2.25)	1.73 (1.78)	2.50 (1.76)	2.27 (2.01)	2.97 (2.25)	4.25 (2.22)	2.28 (1.59)	3.02 (1.84)	26.67 (6.89)

*Notes:* SPG = Schizotypal Personality Group, IR = Ideas of Reference, ESA = Excessive Social Anxiety, OB = Odd Beliefs, UPE = Unusual Perceptual Experiences, EB = Eccentric Behaviour, NCF = No Close Friends, OS = Odd Speech, CA = Constricted Affect, S = Suspiciousness, TSPQS = Total Schizotypal Personality Questionnaire Score.

**Table 2:** Mean (and SD) sensitivity and reaction time (in ms) for the detection of word and emotion as a function of ear of presentation and SPQ group.

	Low SPQ		High SPQ	
	LE	RE	LE	RE
*Word*				
*d*′	1.78 (0.51)	2.36 (0.60)	1.612 (0.49)	2.31 (0.60)
RT	868.81 (156.51)	814.91 (146.89)	863.79 (173.04)	805.26 (140.19)

*Emotion*				
*d*′	2.44 (0.69)	2.02 (0.73)	2.07 (0.70)	1.73 (0.67)
RT	933.53 (164.22)	1002.55 (232.51)	941.19 (195.94)	985.13 (191.46)

## References

[b0005] Aguirre F., Sergi M., Levy C. (2008). Emotional intelligence and social functioning in persons with schizotypy. Schizophrenia Research.

[b0010] American Psychiatric Association (1987). Diagnostic and statistical manual of mental disorders.

[b0015] Bleich-Cohen M., Hendler T., Kotler M., Strous R.D. (2009). Reduced language lateralization in first-episode schizophrenia: An fMRI index of functional asymmetry. Psychiatry Research: Neuroimaging.

[b0020] Borod J.C., Cicero B.A., Obler L.K., Welkowitz J., Erhan H.M., Santschi C. (1998). Right hemisphere emotional perception: Evidence across multiple channels. Neuropsychology.

[b0025] Broks P. (1984). Schizotypy and hemisphere function. II. Performance asymmetry on a verbal divided visual-field task. Personality and Individual Differences.

[b0030] Broks P., Claridge G., Matheson J., Hargreaves J. (1984). Schizotypy and hemisphere function-IV. Story comprehension under binaural and monaural listening conditions. Personality and Individual Differences.

[b0035] Bruder G., Rabinowicz E., Towey J., Brown A., Kaufmann C.A., Amador X. (1995). Smaller right ear (left hemisphere) advantage for dichotic fused words in patients with schizophrenia. American Journal of Psychiatry.

[b0040] Bryden M.P., MacRae L. (1988). Dichotic laterality effects obtained with emotional words. Neuropsychiatry, Neuropsychology, and Behavioral Neurology.

[b0045] Cohen J. (1988). Statistical power analysis for the behavioural sciences.

[b0050] Conn R., Posey T.B. (2000). Dichotic listening in college students who report auditory hallucinations. Journal of Abnormal Psychology.

[b0055] Edgar J.C., Yeo R.A., Gangestad S.W., Blake M.B., Davis J.T., Lewine J.D. (2006). Reduced auditory M100 asymmetry in schizophrenia and dyslexia: Applying a developmental instability approach to assess atypical brain asymmetry. Neuropsychologia.

[b0060] Edwards J., Jackson H.J., Pattison P.E. (2002). Emotion recognition via facial expression and affective prosody in schizophrenia: A methodological review. Clinical Psychology Review.

[b0065] Green M.F., Hugdahl K., Mitchell S. (1994). Dichotic listening during auditory hallucinations in patients with schizophrenia. American Journal of Psychiatry.

[b0075] Hori H., Ozeki Y., Terada S., Kunugi H. (2008). Functional near-infrared spectroscopy reveals altered hemispheric laterality in relation to schizotypy during verbal fluency task. Progress in Neuro-Psychopharmacology & Biological Psychiatry.

[b0080] International Committee of Medical Journal Editors (1991). Statements from the Vancouver group. British Medical Journal.

[b0085] Johns L.C., van Os J. (2001). The continuity of psychotic experiences in the general population. Clinical Psychology Review.

[b0090] Josse G., Tzourio-Mazoyer N. (2004). Hemispheric specialization for language. Brain Research Reviews.

[b0095] Langdon R., Coltheart M. (2004). Recognition of metaphor and irony in young adults: The impact of schizotypal personality traits. Psychiatry Research.

[b0100] Nunn J., Peters E.R. (2001). Schizotypy and patterns of lateral asymmetry on hemispheric language tasks. Psychiatry Research.

[b0105] Oldfield R.C. (1971). The assessment and analysis of handedness: The Edinburgh inventory. Neuropsychologia.

[b0110] Overby L.A. (1992). Perceptual asymmetry in psychosis-prone college students: Evidence for left hemisphere overactivation. Journal of Abnormal Psychology.

[b0115] Phillips L.K., Seidman L.J. (2008). Emotion processing in persons at risk for schizophrenia. Schizophrenia Bulletin.

[b0120] Premkumar P., Ettinger U., Inchley-Mort S., Sumich A., Williams S.C., Kuipers E. (2011). Neural processing of social rejection: The role of schizotypal personality traits. Human Brain Mapping.

[b0125] Raine A. (1991). The SPQ: A scale for the assessment of schizotypal personality based on DSM-III-R criteria. Schizophrenia Bulletin.

[b0130] Rawlings D., Borge A. (1987). Personality and hemisphere function: Two experiments using the dichotic shadowing technique. Personality and Individual Differences.

[b0135] Rawlings D., Claridge G. (1984). Schizotypy and hemisphere function III. Performance asymmetries on tasks of letter recognition and local–global processing. Personality and Individual Differences.

[b0140] Reuter-Lorenz P., Davidson R.J. (1981). Differential contributions of the 2 cerebral hemispheres to the perception of happy and sad faces. Neuropsychologia.

[b0145] Ross E.D., Orbelo D.M., Cartwright J., Hansel S., Burgard M., Testa J.A. (2001). Affective-prosodic deficits in schizophrenia: Comparison to patients with brain damage and relation to schizophrenic symptoms [corrected]. Journal of Neurology, Neurosurgery, and Psychiatry.

[b0150] Sakuma M., Hoff A.L., DeLisi L.E. (1996). Functional asymmetries in schizophrenia and their relationship to cognitive performance. Psychiatry Research.

[b0155] Siever L.J., Davis K.L. (2004). The pathophysiology of schizophrenia spectrum disorders: Perspectives from the spectrum. American Journal of Psychiatry.

[b0160] Sommer I.E.C., Ramsey N.F., Kahn R.S. (2001). Language lateralization in schizophrenia, an fMRI study. Schizophrenia Research.

[b0165] Suzuki A., Usher M. (2009). Individual differences in language lateralisation, schizotypy and the remote-associate task. Personality and Individual Differences.

[b0170] Toomey R., Schuldberg D. (1995). Recognition and judgment of facial stimuli in schizotypal subjects. Journal of Communication Disorders.

[b0180] Woermann F.G., Jokeit H., Luerding R., Freitag H., Schulz R., Guertler S. (2003). Language lateralization by Wada test and fMRI in 100 patients with epilepsy. Neurology.

